# Africa’s businesswomen – underfunded or underperforming?

**DOI:** 10.1007/s11187-023-00792-0

**Published:** 2023-06-17

**Authors:** Charles Ackah, Holger Görg, Aoife Hanley, Cecilia Hornok

**Affiliations:** 1 University of Ghana - ISSER, Accra, Ghana; 2 Kiel Institute for the World Economy, Kiel, Germany; 3 Kiel Centre for Globalization, Kiel, Germany

**Keywords:** Female-owned businesses, Liquidity, Productivity, Supplier credit, Africa, Ghana, D22, J16, L26

## Abstract

While the recent success of *Africa’s ‘Lionesses’* – successful female entrepreneurs – is internationally celebrated, less is known about how liquidity can fuel the success of the *‘Lionesses’* and other businesswomen. Using information from a panel of over 800 male- and female-owned businesses in Ghana (ISSER-IGC survey), we capture a measure of underfunding, in addition to data on supplier credit, equity and other finance sources. Our regressions reveal a female-to-male productivity gap of between − 11 and − 19 per cent, values similar to estimates for other African countries. However, when financial constraints are taken into account, the gender performance gap disappears. Accordingly, female business owners who indicate that *funding is not a problem* are associated with higher productivity than males, all things equal. In a finding new to the literature, our regressions reveal the importance of supplier credit for Africa’s businesswomen.

## Abstract (plain language summary)

300 African Businesswomen in Focus - Suppliers Key to Success. Suppliers to Africa’s businesswomen (e.g. sellers of cloth for garment manufacture) who offer the possibility for delayed repayment, statistically boost the survival of female-owned businesses. *Africa’s* *‘Lionesses’* – exceptional businesswomen – are a comparative rarity, a reason we explore the reasons behind their success. Using data from over 800 Ghanaian businesses from 2011 to 2015, we examine the role of funding in explaining why the majority of businesswomen perform so poorly. Our findings highlight an unusual fact – suppliers to female-owned businesses (e.g. sellers of cloth for manufacture into garments) who offer their female customers the possibility to delay repayments are enormously beneficial in narrowing the gender gap. Targeting tax-cuts towards such suppliers would boost the emergence of future *‘Lionesses’*.

## Introduction

The world has recently witnessed the emergence of successful female business leaders on the continent of Africa. These female business owners include Ethiopian Bethlemen Alemu, director of *SoleRebels* who grew up in a neighbourhood of textile artisans. While such *African Lionesses* are well known and celebrated, Bethlemen Alemu may well be the exception rather than the rule. In many countries, both developed and developing, fewer women than men succeed in starting a business (Kelley et al., 2010). And those businesses that get started by women generally perform worse than their male counterparts.

Access to finance is blamed as an aggravating factor (e.g. McKenzie & Paffhausen, 2019), where women may find it comparatively difficult to obtain funding from formal sources such as banks (e.g. Aristei & Gallo, 2016; Chaudhuri et al., 2020,). However, bank lending is only one aspect of an overall story where funding from family and friends or supplier credit may help to fill the funding gap (e.g. Beck et al., 2008; Boudreaux et al., 2022; Pham & Talavera, 2018).

Whether access to different funding sources helps to explain performance differences for female business owners in developing countries has, to the best of our knowledge, not been investigated to date. Our analysis aims to shed light on this issue. Additionally, our analysis proposes to tie together two strands of research – studies on the *funding* of developing country females (e.g. Maden, 2015; Naguib and Jamali, 2015; Taiwo, 2023) and studies documenting the *performance gap* for these females (Agyire-Tettey et al., 2018; Campos & Gassier, 2017; Langevang et al., 2015; De Mel et al., 2008; Owoo et al., 2019). By estimating an empirical model which takes both funding and performance into account, we can be more certain that any funding deficit for females compared to males is suggestive of credit constraints. Moreover, our Ghanaian data for over 800 male- and female-owned businesses from 2011 to 2015 represents one of the most comprehensive investigations into this issue. To date, work has focussed on more limited data from focus groups or smaller surveys.

Our analysis is prompted by an appeal (e.g. Halkias, 2011; Henry et al., 2016; Verheul et al., 2006) for a multivariate model which controls for confounding covariates (e.g. business sector) that might otherwise skew evidence of a female funding-performance gap. Female business owners dominate in sectors where businesses are traditionally small and not highly efficient. For this reason, Henry et al., (2016) highlight the need for researchers to control for industry sector and apply proper sampling practices.

In sum, our analysis investigates the funding-performance gap for female business owners in Ghana, where we conjecture that – in the absence of credit constraints – business performance (productivity or exporting) should line up with ex ante funding for both males and females.

Our analysis contains a further novelty – namely, the use of firm-level panel data. Longitudinal micro-data is relatively uncommon in Africa, and our database provides a useful exception. Specifically, we use data extracted from the ISSER-IGC enterprise survey collected by Ghana’s Institute of Statistical, Social and Economic Research (ISSER). Additionally, the focus on Ghana has further advantages, a country scoring highly across several metrics for female business owners (Kelley et al., 2010; MIWE, 2022). Ghana ranks second in Africa for female entrepreneurs and in 46th place in the global rankings.^1^ But even in Ghana, female business owners perform worse than their male peers, with businesswomen reporting reduced productivity (Owoo et al., 2019) and sales (Agyire-Tettey et al., 2018).

The granularity of our data allows us to probe aspects of financial constraints and finance sources in a way which has not – to our knowledge – been done to date. Our indicator for financial constraints measures the severity of such constraints (on a scale of 1 to 9), as reported by the enterprises in the survey. In the spirit of the studies emphasising the importance of liquidity, we review differences in financial constraints between female- and male-owned businesses, evaluating how they connect to differences in productivity (for which we use alternative measurements). We apply the data to consider a range of alternative finance sources. We also use export activity as another indicator of firm performance. While there is a small number of empirical studies on gender differences in exporting behaviour (e.g. McClelland et al., 2005), such studies have not looked at the interaction between gender and access to finance as we do in this paper.

Our empirical work uncovers some interesting findings. Female business owners perform worse than their male peers, ceteris paribus, even if businesses in the same industry, city, age and size category are compared – a fact noted in previous studies (e.g. Bardasi et al., 2011; Owoo et al., 2019). In value terms, the female-to-male productivity gap lies in the range of − 11 to − 19 per cent, depending on the productivity measure used – comparable with estimates for other African countries (e.g. Aterido et al., 2011). The gender gap in export propensity is estimated to be roughly − 2 percentage points – a substantial difference, given that the average enterprise in our database exports with a single-digit probability.

Considering constraints in access to finance changes this story. We now find that females that are severely financially constrained perform worse than males, while this is not true for women that do not report financial constraints. In fact, our results indeed show that female business owners reporting that *funding is not a problem* are associated with higher productivity compared to men, all things equal. Interestingly, access to finance is not an issue for male-owned businesses. These results are robust to employing alternative productivity measures, the first lag of the financial constraint variable, or exporting status as the outcome variable.

Moreover, we also find that not all sources of business liquidity are created equal. Female business owners, having a higher recourse to private savings to shore up their liquidity, are associated with reduced productivity. This hints at a worrying possibility – Women relying on their own savings may be forced to do so from a lack of competitive alternatives. On a positive note, we find robust evidence that females sourcing credit from suppliers report higher productivity, all things equal.

We structure our analysis in the following way. We first describe the related literature to help motivate and inform our hypotheses and the methodology we use to test the hypotheses. We then describe our data, introducing the ISSER-IGC panel, and then follow the analysis section before we conclude with some comments on the implication of our findings.

## Gender performance gap – studies and hypotheses

There is a small but well-organised literature reporting the underperformance of female-managed or female-owned businesses in developing countries (e.g. Campos & Gassier, 2017). Generally, the findings of these studies are pessimistic, whereby females perform significantly worse than their male peers. There are a few exceptions to this general rule, but these tend to be sector-specific (See Amin & Islam, 2014). But broadly speaking, at a conceptual-theoretical level, differences in firm performance depending on the gender of the business owner or manager are due to systematically different choices made by males and females. As Croson and Gneezy (2009) argue, women are, on average, much more reluctant than men to engage in competitive behaviour, which may lead to differences in the performance of the firms that they run. However, these differences in choices may be driven by differences in constraints between males and females, which put a limit on female managers’ choices regarding investments, competitive behaviour or risk taking (Campos & Gassier, 2017).

A number of such constraints have been identified in the literature, e.g. underinvestment by female business owners needing to support their families (Fafchamps et al., 2014; McKenzie & Paffhausen, 2019), difficulties in raising external capital (Field et al., 2010; McKenzie, 2017), in leveraging family business networks (Aterido & Hallward-Driemeier, 2011), in women being treated differently by investors (Kanze et al, 2018) or suffering from poorer education (Islam & Amin, 2016; Islam et al, 2019). Generally, the consensus view is that females face more severe constraints on accessing finance from different sources.^2^

Empirical studies on the gender funding gap have tended to focus on an evaluation of *bank* lending (Aterido et al., 2013). But a parallel literature has highlighted the role of liquidity shocks (not exclusively credit shocks) on the performance of enterprises in developing countries (McKenzie, 2017; Rotemberg, 2019). We now proceed to review these studies, identifying two main ideas which have not been satisfactorily answered to date. First, does the empirical evidence point towards a gender performance gap, even when the idiosyncrasies of female- and male-owned businesses are considered? And second, is there evidence that additional liquidity would help to mitigate this problem?

### The productivity premium – returns to liquidity for female- and male-owned businesses

There exists a relatively large literature on how access to finance affects the growth of small and medium-sized enterprises, where growth is measured either as sales growth or employment growth (Fafchamps and Schündeln, 2013; Fowowe, 2017; Ayyagari et al., 2021). The gender performance gap is well documented, with some researchers reporting statistical differences in male- and female-owned businesses for employment size (Bardasi et al., 2011; Chaudhuri et al., 2020), productivity (Aterido & Hallward-Driemeier, 2011; Chaudhuri et al., 2020), growth (Belitski & Desai, 2021; Chaudhuri et al., 2020; Coad & Tamvada, 2012) or export participation rates (Presbitero et al., 2014). But, in the absence of highly granular data, it is difficult to grasp the severity of the problem. If, for example, women are overrepresented in low-paying, informal or traditional sectors – characterised by low productivity and earnings (see Klapper & Parker, 2011) – then controlling for such sectoral information might well cause the productivity gap to disappear altogether.

In our paper, we focus on a performance measure that has received less attention in the context of financial constraints: productivity. Productivity measures how efficiently inputs are transformed into output, and, as such, it is a prominent performance measure of a manufacturing enterprise. Sales or employment capture the size of a company, but not necessarily production efficiency, as larger companies are not necessarily more productive. Our focus on productivity also draws on a large literature on heterogeneous firms (originating from the seminal paper of Melitz, 2003), which studies how productivity determines the success of manufacturers in domestic and international markets.

Having established (or otherwise rejected) the possibility of a gender productivity gap, the next step is to analyse whether funding (loans or other liquidity sources) makes any difference in mitigating this gap. If, as argued above, gender-related constraints on access to finance exist, then female owners/managers may make systematically different decisions due to the unavailability of adequate funding to their firms. If this is the case, then such financial constraints may explain part, if not all, of the female performance gap.

While the literature generally agrees that female-owned businesses perform worse than their male-owned peers, the empirical evidence for credit constraints is more mixed. In a recent study, Chaudhuri et al. (2020) use data from business owners in India, splitting the coefficient of loan denial into an *endowment* component (female business owners constrained to exhibit the same endowments as their male peers) and a *characteristics* component (lenders apply the same criteria to females as males).^3^ Their study reveals that the higher rejection rates on credit applications from businesswomen are not a symptom of credit constraints. Rather, the higher rejection rates for female applicants are underpinned by quality differences in the loan application. The characteristics of female loan applicants are so qualitatively different – negatively so – from their male peers that the higher rejection rates cannot be blamed on gender discrimination. Other studies come to a similar conclusion – Female loan applicants report higher rejection rates due to the inferior quality of their applications for finance rather than any underlying, gender-based discrimination (Aterido & Hallward-Driemeier, 2011; Aterido et al., 2013; Bardasi et al., 2011).

But other studies contradict these findings. At least three of the most cited of these analyses reveal substantial evidence for gender-biased credit constraints (Aristei & Gallo, 2016; Muravyev et al., 2009; Presbitero et al., 2014). Most recently, Aristei & Gallo (2016) uncover evidence of gender discrimination when business owners apply for credit. Their data covers 28 transitional European countries. Here, the differences in denial rates are not due to covariates used in their regressions but to unexplained sources of variation (factors not picked up in their estimations). Similarly, Muravyev et al. (2009) also pick up variation in the error term consistent with a regime of gender-based credit rationing. Finally, Presbitero et al. (2014) uncover evidence of credit constraints, employing data for around 360 borrowers across 3 Caribbean countries.^4^

From the studies reviewed so far, we find that evidence is almost evenly split for and against gender discrimination in credit markets. But there is a caveat connected to the existing work, its heavy emphasis on loans applied for and loans rejected. The literature is largely silent on the overall liquidity position of female business owners. Moreover, evidence by Bardasi et al. (2011) suggests that the demand for loans by female borrowers is not accurately measured. This is due to the *discouraged borrower effect*, where female business owners in developing countries may be reluctant to apply for a bank loan or line of credit, anticipating a rejection. Evidence for this discouraged borrower effect is corroborated by Gonzalez-Uribe & Leatherbee (2018).^5^ These studies highlighting the discouraged borrower effect hint at the wisdom of widening the definition of funding to include other sources of liquidity. This is because studies focussing on bank loans – due to the discouraged borrower effect – are likely to underestimate the real liquidity problem.

From this discussion, we can formulate the following hypothesis.

- **H1**: A gender-related productivity gap can be (at least partly) explained by differences in access to funding between female- and male-owned businesses.

### Reliance on personal savings as a litmus test for liquidity-constrained businesswomen

There are two further hypotheses we can investigate with our data. The first is connected with the idea of broadening liquidity sources to include other sources of liquidity apart from bank loans. Specifically, Beck et al. (2008) have argued that business owners in the middle- and low-income countries have very different funding possibilities to their peers in Germany or the UK. There are more commonly used alternatives to formal working capital loans, e.g. supplier credit. With respect to supplier credit, Beck et al. examine the financing patterns for firms across 48 countries, including many developing countries. Their findings build on evidence from the World Business Environment Survey, administered by the World Bank. Supplier credit represents the second-most important source of finance for small firms in developing countries, after bank credit. Meanwhile, in a recent study using data for entrepreneurs in Zambia, the role of supplier credit in reducing information asymmetries is clear (Boudreaux et al., 2022). Suppliers working close to the entrepreneur can gauge the individual’s social capital, arguably better than a bank.

This idea of formal vs informal finance is further developed by Pham & Talavera (2018) using data across the size spectrum for Vietnam. On the basis of their estimations, they conclude that businesswomen are *more successful* in obtaining loans than their male peers. Additionally, businesswomen enjoy the privilege of reduced interest rates. Pham and Talavera attribute the stronger loan performance of Vietnamese businesswomen to a buoyant supply of informal finance (loans from friends and relatives). The Pham and Talavera study underpins the importance of viewing bank finance as only one component in a wider and richer picture.

As Pham & Talavera (2018) have highlighted, supplier credit represents a widely used way to boost short-term liquidity – a cheap and less complex alternative to overdraft finance. The payback period is short (typically a month, in the case of Ghana), but the loan is interest free. Importantly, the entrepreneur can bridge the time between procurement of materials, working these materials to a final product which can be sold for cash. Unlike banks (which are highly regulated and subject to public scrutiny), suppliers have much latitude in the terms they offer to their business customers (Fafchamps, 2000). Additionally, as noted earlier, a recent study using data on 1971 entrepreneurs in Zambia has highlighted the role of suppliers in providing credit to developing country entrepreneurs (Boudreaux et al., 2022). Specifically, suppliers are in an excellent position to gauge the creditworthiness of their clients through repeated transactions.

For businesses, it is a good thing to be able to source funding from a variety of providers (banks, investors and suppliers) as it widens the set of funding possibilities. The portion of funding from these providers can vary from firm to firm. On one aspect, there is a universal consensus – A disproportionate reliance on cash savings by any group suggests a deficit in the provision of formal finance (Guérin, 2006; Loaba, 2022). Moreover, Guérin has argued that the reliance of females on informal finance is a consequence of gender inequalities, an over-reliance that can perpetuate further inequality. Meanwhile, Loaba has demonstrated that women are more reliant on informal sources of finance than males. One glimmer of hope is offered by newer technological possibilities (e.g. mobile money), helping females to sidestep the perceived shortfall in bank credit. But many funding sources, so long as they are competitively priced (e.g. supplier credit), can be used to help the female entrepreneur to expand her market share. In this way, she can improve her productivity. Indeed, the usefulness of bank credit may be overvalued. As has been shown using data for India, excessive reliance on bank credit can point to cashflow problems within a firm (Satpathy et al., 2017). For this reason, it is not easy to propose an ideal split between formal vs informal sources of liquidity. But broadly speaking, we expect that a widened set of funding possibilities can help an entrepreneur to boost the productivity of her firm, reducing the gender performance gap.

- **H2**: Access to other informal sources of finance mitigates the gender performance gap.

### Exporting as an alternative performance metric

Our final hypothesis concerns itself with exporting, another performance metric – apart from productivity. We believe that export participation is also an important measure of firm performance. For one, following the paper of Bernard and Jensen (1999), a vast literature on heterogeneous firms and international trade documents that productivity and exporting correlate strongly positively. Manufacturers need to achieve a certain level of productivity to enter export markets, while export participation can improve productivity further. Second, the export participation of African manufacturers is of great policy importance. The exports of most African countries remain dominated by primary products, while manufacturing exports are historically low and likely to remain so for various reasons (Wood and Mayer, 2001). This hinders the continent’s economic development. Consequently, policymakers in many African countries – including Ghana – have a strong interest in learning about the drivers of manufacturing exports.

Although, in our data, exporting is a small number phenomenon, making it difficult to pick up empirically, exporting is an activity often pushed by policymakers in developing countries. Exporting to other developing or even developed countries can help indigenous businesses to buffer against demand shocks in their home country, broaden their customer base and motivate them to redouble their efforts to reach the world technology frontier in order to remain competitive with a widened set of competitors. The benefits of exporting are well documented (e.g. Girma & Görg, 2022; Van Biesebroeck, 2005).

Furthermore, export activity is often used as an alternative measure of firm performance, focusing on the international engagement of a firm. The overwhelming evidence shows that exporting firms are more productive than non-exporters (e.g. Wagner, 2019). A small number of studies have looked at gender differences in export performance, showing that female-owned firms, on average, are less export-oriented than their male-owned counterparts (Manolova et al., 2002; McClelland, 2004; McClelland et al., 2005). One explanation given for these performance differences is restricted access to funding for female entrepreneurs (McClelland et al., 2005).

But if female-owned businesses find it comparatively more difficult to access export markets due to a lack of funding, then this bias needs to be corrected. We conjecture that female-owned businesses – lacking adequate liquidity – find it more difficult to contest export markets. No study – to our knowledge – has in detail examined exporting in the context of the interaction of gender and liquidity constraints.

This hypothesis is expressed as follows.

- **H3**: There is a gender export gap, which is driven by underfunded females.

Before moving on to the main empirical section, we first describe our data.

## The Ghanaian ISSER-IGC panel

We recall our initial research question to (1) investigate Ghana’s gender performance gap and (2) ascertain whether this performance gap explains any differences in the perceptions of business owners of both genders that they are underfunded.

To address these questions, we use data from the ISSER-IGC survey. This survey of micro, small and medium-sized manufacturing enterprises in Ghana is administered by the Institute of Statistical, Social and Economic Research (ISSER) based at the University of Ghana and funded by the International Growth Centre (IGC). In terms of timing, questionnaires were distributed in August/September 2016. The survey elicited information on the characteristics of business owners and their businesses for five consecutive years (2011 to 2015, inclusive).^6^

The sample frame adopted for the questionnaire was extracted from the first phase of the Ghana Integrated Business Establishment Survey (IBES). The latter represents an economic census of non-household enterprises conducted by the Ghana Statistical Service (GSS) from 2014 to 2015. To undertake the survey, the sample frame was extracted from the universe of manufacturing micro, small and medium-sized enterprises (MSMEs) located in the cities of Accra, Tema, Kumasi and Sekondi-Takoradi. These cities represent the main industrial clusters of Ghana. To help completeness, the data also includes firms from Ghana’s informal sector. From the IBES, all manufacturing MSMEs located in the four cities were selected. This amounted to 1244 firms in total. The interviewers conducting the survey encountered a reasonable response rate. However, there was some sample attrition. This was due to firms declining to participate (73 firms), business closure (55 firms) and failure to locate the business (231 firms). To sum up, altogether, 880 firms completed the questionnaire, corresponding to a 70 per cent response rate.

The sampled firms operate in 20 different two-digit manufacturing industries, applying the International Standard Industrial Classification (ISIC) Revision 4 classification. Table 1 illustrates the geographic and sectoral breakdown of these firms, where industries are grouped into four categories acknowledging the strong concentration of the firms in a few industries. The Accra and neighbouring Tema area account for about half the firms. Sekondi-Takoradi, also on the coastline, represents about an eighth of the firms. The remaining firms are located further inland, in the city of Kumasi. In terms of the business sector, the overwhelming majority of the businesses are active in the Textiles and Clothing sector, followed by Wood Processing and Food and Beverages.

**Table 1:** Number of firms by sector and location

	Location of enterprise					Gender of business owner		
Industry group	Accra	Tema	Kumasi	Sekondi-Takoradi	Total	Male	Female	Total*
Food and beverages	40	20	41	16	117	38	78	116
Textiles and clothing	198	35	218	62	513	228	284	512
Wood processing	59	22	84	14	179	164	3	167
Other manufacturing	28	3	31	9	71	61	6	67
Total	325	80	374	101	880	491	371	862

Note: The sample of firms in the right part of the table excludes 18 enterprises that are either owned by the state or do not report the gender of the owner

Klapper & Parker (2011) noted the over-representation of females in the informal sector. Alternatively, in sectors with the least potential for growth and profits, while the food and beverages sector is dominated by female business owners (67 per cent), Ghana’s main industry, textiles and clothing, exhibits almost equal proportions of male and female business owners, with females comprising 55 per cent of this sector. Altogether, 43 per cent of the business owners in our sample are females.

We continue with the discussion of the most important variables in our empirical analysis. A systematic description of all variables used is presented in Table 2. Basic descriptive statistics are shown in Table 3.

**Table 2:** Description of variables used

Variable	Description	Source/notes
ID	Enterprise ID	Original variable, ISSER-IGC panel
Year	Calendar year (2011–2015)	Original variable, ISSER-IGC panel
Industry	Industry code ISIC rev. 4 (categorical, 1–24)	Original variable, ISSER-IGC panel
Sector	Broad industry (categorical, 1–4)	*industry* grouped into four larger categories (food and beverages, textiles and clothing, wood processing, other manufacturing)
Location	City in which the enterprise is located (categorical, 1–4)	Original variable, ISSER-IGC panel
Female	Primary owner of enterprise is female (binary)	0: male; 1: female
Age	Age of enterprise (years)	*year* – year of initial production (as reported in the survey)
Size	Size of enterprise in terms of employment (categorical, 0–2)	0: micro (1–5 employees); 1 small (6–19 employees); 2 medium (20 + employees)
Exporter	Enterprise exports some of its production output (binary)	0: no export; 1: export
Foreign	At least 10% of the enterprise is owned by a foreign owner (binary)	0: not foreign-owned 1: foreign-owned
tfp_ac	Total Factor Productivity of enterprise (logarithm)	Estimated by the Ackerberg-Caves-Frazer (ACF) estimator
tfp_lp	Total Factor Productivity of enterprise (logarithm)	Estimated by the Levinsohn-Petrin estimator
tfp_wr	Total Factor Productivity of enterprise (logarithm)	Estimated by the Wooldridge estimator
Finance constraint (FC)	Access to finance as business constraint (rank variable, 1–9, higher indicates more severe constraint)	The variable is based on the survey question, “Please rank the following nine obstacles in terms of their importance to the enterprise’s operations: access to finance, taxation, customs and regulation, security, bribery/informal payments, access to land, access to electricity, access to other infrastructure, market access.”
FC categories	Access to finance as business constraint (categorical, 0–2)	It is generated from the finance constraint variables. It takes value 0 if the finance constraint is 1, 2 or 3 (low), value 1 if the finance constraint is 4, 5 or 6 (medium), and 2 if the finance constraint is 7, 8 or 9 (high)
FS bank loan	Bank loan from formal institutions (% of working capital)	FS variables are based on the survey question, “What percentage of the enterprise’s working capital was obtained from the following sources?”
FS own resources	Personal savings and retained earnings (% of working capital)	
FS friends and relatives	Loan from friends and relatives (% of working capital)	
FS suppliers credit	Suppliers credit (% of working capital)	
FS equity and bond	Issuance of equity and bonds (% of working capital)	
FS other	Other finance sources (% of working capital)	
Variables used in TFP estimation		
Y	Value added of production (2006 cedis)	Generated as the value of production output minus the value of raw materials used in production (both from the ISSER-IGC panel, deflated by *PPI*)
L	Number of workers (both production and non-production)	Original variable, ISSER-IGC panel
K	Estimated resale value of capital (land, buildings, machinery and equipment) (2006 cedis)	Original variable, ISSER-IGC panel, deflated by *PPI*
PPI	Producer Price Index (2006 = 1) for the manufacturing sector in Ghana	Ghana Statistical Services

**Table 3:** Descriptive statistics of key variables

Variable	Total			Female-owned			Male-owned		
	Obs	Mean	Std. dev	Obs	Mean	Std. dev	Obs	Mean	Std. dev
Female	4310	0.430	0.495	1855	1.000	0.000	2455	0.000	0.000
Age	4105	13.621	9.644	1760	13.335	8.859	2345	13.836	10.190
Size	4207	0.766	0.603	1824	0.737	0.575	2383	0.788	0.622
Exporter	3548	0.035	0.184	1549	0.019	0.138	1999	0.048	0.213
Foreign	4310	0.017	0.131	1855	0.008	0.090	2455	0.024	0.154
tfp_ac	3891	7.570	1.254	1674	7.414	1.101	2217	7.688	1.346
tfp_lp	3891	8.139	1.294	1674	7.904	1.165	2217	8.317	1.357
tfp_wr	3891	8.071	1.468	1674	7.825	1.352	2217	8.257	1.523
Finance Constraint (FC)	4233	7.250	1.877	1820	7.219	1.887	2413	7.273	1.869
FC categories	4233	1.701	0.581	1820	1.700	0.581	2413	1.701	0.581
FS bank loan	4206	3.011	12.726	1822	2.142	10.226	2384	3.675	14.313
FS own resources	4206	80.713	33.212	1822	81.049	33.205	2384	80.456	33.222
FS friends and relatives	4206	3.369	15.133	1822	2.953	13.792	2384	3.688	16.079
FS suppliers credit	4206	5.345	16.202	1822	5.113	16.090	2384	5.523	16.288
FS equity and bond	4206	2.357	12.686	1822	1.965	11.631	2384	2.657	13.431
FS other	4206	5.204	19.745	1822	6.778	22.460	2384	4.002	17.294

Note: The sample excludes observations of enterprises that are either owned by the state or do not report the gender of the owner

### Calculating productivity

We measure productivity as total factor productivity (TFP) using a regression framework to estimate production functions.^7^ As we have signalled in the ‘Introduction’, one innovation of our analysis is the provision of alternative measures of TFP, allowing us to choose the TFP candidate which offers the most reliable estimates. Given the focus on productivity differences in our paper, we now outline our various TFP models using alternative variants of the workhorse proxy variable estimation methods (also called as control function approach) first proposed by Olley & Pakes (1996). An advantage of estimating total factor productivity (TFP) in several different ways is to help raise confidence in the point estimates. On balance, our favoured estimation method is the most recent Ackerberg–Caves–Frazer, short ACF method (Ackerberg et al., 2015).

Although these estimation methods are now considered standard in the literature, we describe them here briefly. The total factor productivity (TFP) of a firm in a given year is measured as the residual from a production function estimation. We assume a Cobb–Douglas production function of the value-added output with two inputs – capital and labour – and standard Hicks-neutral technological change. Formally,

1 $${y}_{it}={{\alpha }_{k}k}_{it}+{{\alpha }_{l}l}_{it}+{\omega }_{it}+{\varepsilon }_{it}$$

where ${y}_{it}$ is value-added output for firm *i* in year *t*, ${k}_{it}$ and ${l}_{it}$ are capital and labour, respectively, ${\omega }_{it}$ is the unobserved total factor productivity and ${\varepsilon }_{it}$ is an idiosyncratic error term. All variables are in logarithm. Value added is obtained as the value of gross output less the cost of raw materials, capital is measured as the replacement cost of capital items including land, buildings and machinery, and labour is the number of workers, as reported by firms in the survey. We deflate all nominal values to 2006 Ghanaian *Cedis* using the manufacturing producer price index from the Ghana Statistical Service.^8^

A well-known challenge in estimating production functions is that input use is not independent of current productivity. The productivity term ${\omega }_{it}$ is unobserved by us and hence becomes part of the error term in the estimation: ${u}_{it}={\omega }_{it}+{\varepsilon }_{it}$. Firms, however, can obtain information on their current productivity and adjust their contemporaneous input use accordingly. This generates a positive correlation between the input variables and the error term ${u}_{it}$, leading to biased estimates when (1) is estimated by OLS. Specifically, OLS coefficient estimates become upward biased for the labour input and downward biased for capital (Olley & Pakes, 1996). To overcome this problem, Olley and Pakes (OP) propose a two-step control function estimation procedure, which was subsequently improved by Levinsohn & Petrin (2003) and Ackerberg et al. (2015). These methods are essentially a proxy for productivity by observable variables (investment or material use). In addition, Wooldridge (2009) proposes a potentially more efficient, one-step estimation procedure that yields the Levinsohn-Petrin estimator.^9^

This paper applies three of the above estimation methods to generate total factor productivity: Levinsohn-Petrin (LP), Wooldridge and Ackerberg-Caves-Frazer (ACF).^10^ Our preferred estimation method is, however, the most recent ACF procedure. Ackerberg et al. (2015) show that, due to functional dependence, it is generally not possible to identify the labour coefficient in the first step of the estimation procedure, which the OP and LP methods do. Labour use is namely functionally dependent on the other variables that are included in the first-stage regression to proxy for productivity. Consequently, the ACF procedure estimates both the capital and labour coefficients in the second step.

**Fig. 1:**
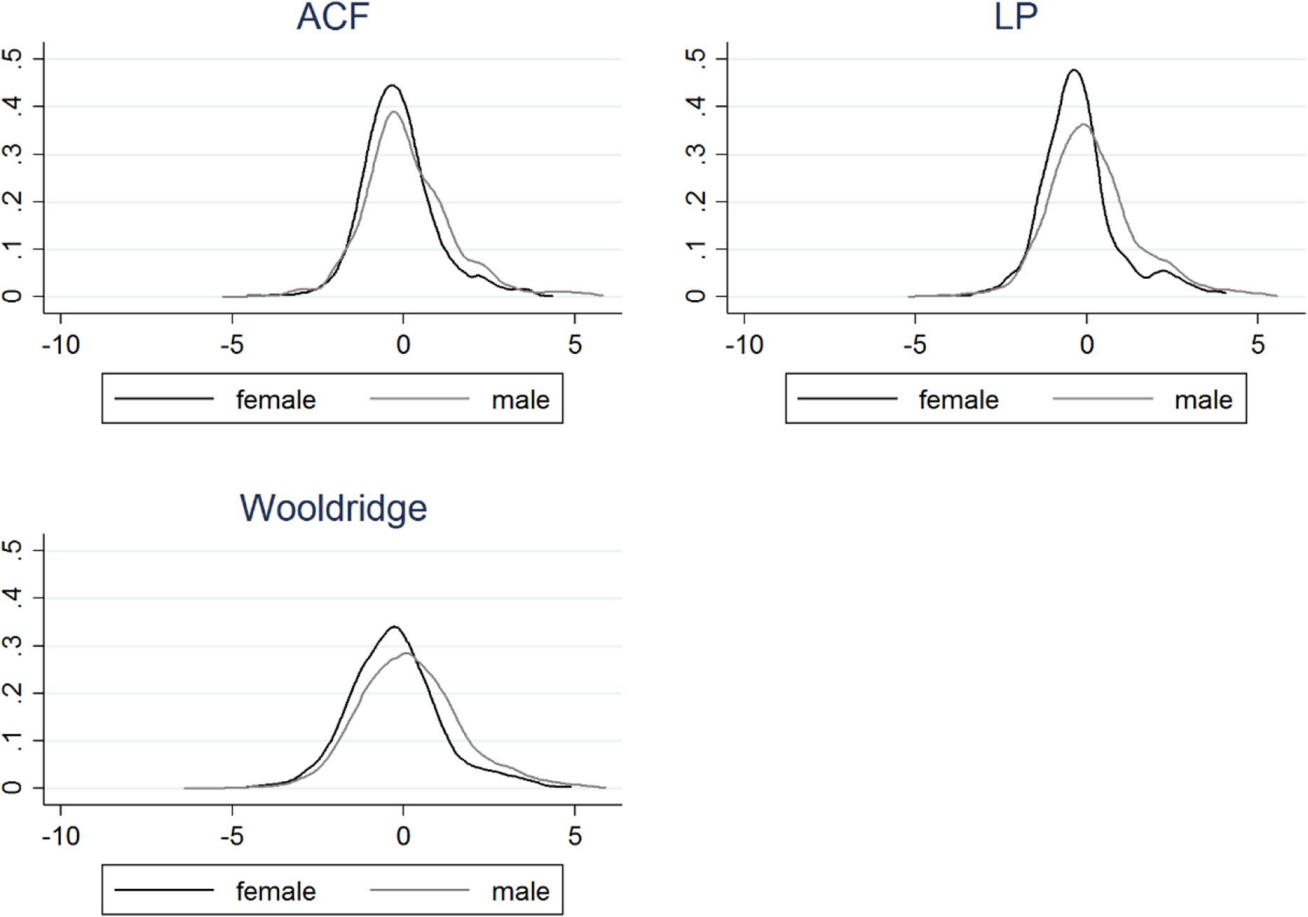
Kernel density estimates for the TFP variables

The TFP measures we obtain as a result of these estimations are sufficiently similar, as indicated by the pairwise correlations of around 0.9 (Table 4). After netting out industry means from the TFP variables, we also plot the TFP distributions in Fig. 1 for male and female business owners, respectively. The kernel density estimates look visually similar for all TFP methods used, with the exception of Wooldridge. Of course, a simple visual comparison of distributions cannot consider other potential differences between these two groups (for example, firm age). Nor do they allow us to infer causality. We, therefore, turn to a more formal modelling of the relationship between gender, funding sources and performance below.

**Table 4:** Pairwise correlation coefficients between key variables

		Female	Age	Size	Exporter	Foreign	tfp_ac	tfp_lp	tfp_wr	FC
Female	rho	1								
	N	4330								
Age	rho	− 0.024	1							
	N	4125	4125							
Size	rho	− 0.0449***	0.0914***	1						
	N	4226	4021	4226						
Exporter	rho	− 0.0749***	0.0091	0.1186***	1					
	N	3563	3412	3523	3563					
Foreign	rho	− 0.0613***	− 0.0599***	0.1018***	0.0388**	1				
	N	4330	4125	4226	3563	4330				
tfp_ac	rho	− 0.1071***	− 0.0042	0.0136	0.0436**	0.0147	1			
	N	3905	3743	3870	3317	3905	3905			
tfp_lp	rho	− 0.1579***	0.0283*	0.1659***	0.0815***	0.0657***	0.9619***	1		
	N	3905	3743	3870	3317	3905	3905	3905		
tfp_wr	rho	− 0.1459***	0.0278*	0.1600***	0.0823***	0.0673***	0.8464***	0.8878***	1	
	N	3905	3743	3870	3317	3905	3905	3905	3905	
FC	rho	− 0.0145	− 0.0242	− 0.0552***	− 0.0124	− 0.1075***	− 0.0289*	− 0.0318**	− 0.0419***	1
	N	4253	4048	4210	3552	4253	3894	3894	3894	4253

Note: Pairwise correlation coefficients and the corresponding sample sizes. The sample size varies between variable pairs due to varying data availability

^*^Significant at 10%

^**^Significant at 5%

^*^Significant at 1%

### Exports

As reported in our literature section, firm-level exporting is an under-researched performance metric in many developing countries. Fortunately, the ISSER-IGC panel includes information on the firm’s export engagement. Specifically, the questionnaire asked respondents to report the share of output that is exported annually (export intensity). Based on this continuous measure, we generate an export dummy for the firm’s export status in any given year. In our sample, only 3.5 per cent of the firm-year observations are exporters, reflecting low export incidence in this representative sample of firms (Table 3). As unconditional correlations in Table 4 reveal, it is mainly the larger and more productive enterprises which sell abroad. Low export incidence is, therefore, not uncommon in a sample of MSMEs.

### Underfunding

One of our most important variables is our proxy for underfunding – the extent to which respondents of both sexes report that lack of access to finance represents a serious obstacle to their business operations.

Why do we need to consider funding and performance in the same empirical model of underfunding? The reason has to do with the concept of credit constraints. Credit constraints only hold sway when funding is denied to an entrepreneur on the basis of reasons other than standard investment criteria. And standard investment criteria – rather than any other criteria – is what underpins the extension of credit from banks, equity investors and other creditors (e.g. suppliers). Precisely, this is the problem making it hard to identify credit constraints – the absence of visible performance metrics for a firm. Because of this, investors can plausibly argue that any applicant group (e.g. females or marginalised applicants from certain religious, ethnic or socio-economic backgrounds) are excluded on the basis of market criteria. However, all things equal, if the performance of marginalised applicants is similar to those of applicants from the non-excluded group, this argument no longer holds and these female applicants may well be credit constrained. Accordingly, the case for credit-constrained females hinges on the business performance of these females since it must be viewed through the lens of the lender.

Since banks (and other investors) should be strictly guided by market criteria awarding or declining an entrepreneur’s application for credit, the entrepreneur’s performance metrics (e.g. productivity or exporting) are key signals of the strength of the entrepreneur’s enterprise and accordingly whether the investment can be repaid, or not. Where such performance metrics are not easily observed (e.g. total factor productivity cannot be obtained as a back-of-envelope calculation), research can still empirically calculate such measures to shed light on whether an entrepreneur is underfunded or not.

In the ISSER-ICG panel, respondents were asked to rank obstacles to the firm’s operations across the years 2011–2015. The obstacle rank variables that we generate from the responses assume integer values between 1 and 9, where 9 indicates the highest and 1 is the lowest importance. ‘Access to finance’ is one of the obstacles on the list. It is also one of the obstacles where male and business owners report such differences in the severity of this problem that we must reject the null of equal distribution in the breakdowns of responses. To illustrate the severity of access to finance as a problem for female business owners, Table 5 supplies the results of Wilcoxon rank-sum tests that compare the distributions of male and female rankings for three obstacles impacting the day-to-day running of the business, (1) access to finance, (2) taxation and 3) market access. The test reports a statistically significant difference between male and female rankings of access to finance. Specifically, female-owned firms are significantly more likely than male-owned ones to designate access to finance as important. This suggests that this obstacle deserves further investigation within a regression framework.

**Table 5:** Wilcoxon tests for key business constraints

	Access to finance	Taxation	Market access
	H0: equal distributions		
Z	− 2,0130	− 0,3310	0,4370
Prob >|z|	**0,0441**	0,7409	0,6625
Pr (male > female)	0,4820	0,4970	0,5040

Note: Before running the tests, we removed the industry means from the business constraint variables to ensure the results are not driven by industry differences in the ratio of male-to-female firms. Values in bold refer to significance at the 95 percent level of confidence

For further analysis, we define a financial constraint (FC) variable on the basis of the obstacle rank for ‘access to finance’. Variable FC, therefore, takes integer values between 1 and 9, where a higher value means more serious finance constraints perceived by the enterprise.

Since financial constraints pose a particular problem for female business owners, the next step is to delve into funding patterns for both genders – how these breakdowns can be linked to a gender funding gap. One point worth exploring in this context is the sources of funding used by male and female business owners – providing a possible intuition for a gender funding gap. The data provides information on a number of different funding sources (FS), namely own resources, loans from friends and relatives, supplier credit, and equity and bond finance (Table 2). Table 6, which reports correlations between the FC variable and the use of different finance sources, illustrates that female business owners who receive a loan are disproportionately less likely (versus their male peers) to report severe financial constraints. The same intuition applies to businesswomen who receive suppliers’ credit.

**Table 6:** Correlations between financial constraint and different finance sources

	Bank loan	Own resources	Friends and relatives	Suppliers credit	Equity and bond	Other
Female owned (*N* = 1812)						
Correlation	− 0,126	0,116	0,055	− 0,137	− 0,008	− 0,046
Significance	0,000	0,000	0,019	0,000	0,729	0,052
Male owned (*N* = 2383)						
Correlation	0,013	0,067	− 0,008	− 0,038	− 0,045	− 0,061
Significance	0,542	0,001	0,701	0,067	0,027	0,003
Total (*N* = 4195)						
Correlation	− 0,035	0,088	0,017	− 0,080	− 0,030	− 0,054
Significance	0,022	0,000	0,260	0,000	0,052	0,001

Note: Correlation coefficients and their significance levels between the use of different finance sources (FS) and the finance constraint (FC) variable. Finance source variables are percentages of working capital financed from a given source. A negative correlation means that firms that report to have more serious problems with access to finance use less of the given finance source. Own resources = retained earnings + personal savings. Other is a residual category, which also includes loans from moneylenders

Although male business owners also seem to value suppliers’ credit in helping to mitigate the perception of underfunding, the correlation is higher for females. As we indicated before, suppliers’ credit represents a key source of liquidity for many developing-world businesses (Beck et al., 2008). Similarly, *both* male and female business owners who dig into their own resources to fund their businesses appear to experience difficulties in tapping appropriate finance. However, the correlation for supplier credit is higher for businesswomen. Finally, male business owners able to source equity finance are far less troubled by underfunding issues. However, because the numbers involved are very small (In 2015, for example, there were only 31 male and 12 female businesses that financed some of their working capital through equity or bond), it is difficult to do more than remark on the potential uplift for these more sophisticated forms of finance, in helping Africa’s business community to scale up their business capacity.

Summing up, both lender and supplier credit appear to attenuate the problem of underfunding for businesswomen. The bivariate correlations above can help to shape our expectations about the role of credit supply and gender. We now embark on a fuller examination of funding using a regression framework which relates underfunding to gender, firm performance and business metrics for the business owner respondents.

## Gender performance gap and liquidity constraints

We first proceed to our main regressions, exploring the role of gender differences in productivity. First, we document the female-to-male productivity gap for Ghanaian MSMEs. Observing these firms for a 5-year annual panel (2011–2015) allows us to estimate the following regression equation using pooled OLS:

2 $${tfp}_{it}={\beta }_{1}{\mathrm{female}}_{i}+\gamma {X}_{it}+{\delta }_{s}+{\delta }_{l}+{\delta }_{t}+{\varepsilon }_{it}$$

The dependent variable is the logarithm of total factor productivity (*tfp*) of enterprise *i* in year *t*. We use either of the three alternative productivity measures as the dependent variable. Our preferred measure is *tfp_ac*, while the remaining two are reported for robustness. The variation in productivity is explained by a dummy denoting whether the primary business owner is female. Apart from the female ownership dummy, we also include several other enterprise characteristics in vector X. Industry, location and time effects are also included in the δ terms.

The time-constant binary variable female takes the values of 1 and 0 for enterprises with female and male primary owners, respectively. (A few state-owned enterprises are excluded from the sample.) The gender productivity gap is captured by the parameter ${\beta }_{1}$, measuring the difference in productivity for female-owned relative to male-owned enterprises, having controlled for the characteristics of the enterprise and other observables. Controlling for this vector of covariates is key to our estimation strategy since these observable characteristics of the enterprise may influence productivity and, at the same time, correlate with the gender of the owner. Covariates we consider include the age and the size of the enterprise, a binary variable denoting foreign ownership, the enterprise’s industry s (2-digit NACE) and location l, as well as year effects common to all enterprises to remove macro trends. We estimate (2) with pooled OLS and robust standard errors.

We now turn to the results for our estimation of the gap in female-entrepreneur TFP and perceived financial constraints (Table 7).

**Table 7:** Gap in female-entrepreneur TFP and perceived financial constraints

Dependent variable:	tfp_ac	tfp_ac	tfp_ac	tfp_lp	tfp_lp	tfp_lp	tfp_wr	tfp_wr	tfp_wr
	(1)	(2)	(3)	(1)	(2)	(3)	(1)	(2)	(3)
Female	− 0.118**	− 0.117**	0.407**	− 0.207***	− 0.207***	0.347*	− 0.212***	− 0.212***	0.293
	(0.0501)	(0.0501)	(0.181)	(0.0508)	(0.0509)	(0.185)	(0.0509)	(0.0509)	(0.187)
Age of firm	− 0.00478**	− 0.00475**	− 0.00438*	− 0.00298	− 0.00295	− 0.00257	− 0.00251	− 0.00247	− 0.00212
	(0.00225)	(0.00225)	(0.00225)	(0.00226)	(0.00226)	(0.00226)	(0.00227)	(0.00227)	(0.00226)
Firm size categories (benchmark is micro)									
Small	− 0.0707	− 0.0739	− 0.0777	0.153***	0.151***	0.147***	0.173***	0.170***	0.166***
	(0.0506)	(0.0506)	(0.0505)	(0.0506)	(0.0505)	(0.0505)	(0.0506)	(0.0505)	(0.0505)
Medium sized	0.0715	0.0637	0.0659	0.707***	0.701***	0.703***	0.763***	0.755***	0.757***
	(0.0918)	(0.0912)	(0.0907)	(0.0959)	(0.0953)	(0.0947)	(0.0974)	(0.0968)	(0.0962)
Foreign	− 0.00609	− 0.0341	0.0419	0.137	0.114	0.195	0.179	0.150	0.223
	(0.167)	(0.166)	(0.169)	(0.162)	(0.161)	(0.165)	(0.167)	(0.165)	(0.169)
Lagged FC		− 0.0148	0.0179		− 0.0120	0.0226		− 0.0154	0.0161
		(0.0125)	(0.0174)		(0.0127)	(0.0174)		(0.0128)	(0.0176)
Female × lagged FC			− 0.0714***			− 0.0756***			− 0.0688***
			(0.0240)			(0.0246)			(0.0248)
Observations	2917	2917	2917	2917	2917	2917	2917	2917	2,917
*R*-squared	0.090	0.090	0.093	0.150	0.150	0.153	0.350	0.350	0.352

Note: OLS estimation with industry, location and year effects. The dependent variables are different TFP estimates in logarithm: tfp_ac stands for the Ackerberg-Caves-Frazer estimate, tfp_lp for the Levinsohn-Petrin estimate and tfp_wr for the Wooldridge estimate. Firm size categories are micro (0–5 employees), small (6–19 employees) and medium (20 + employees). Robust standard errors in parentheses

^***^p < 0.01

^**^p < 0.05

^*^p < 0.1

We report a statistically significant and negative female-to-male productivity gap, within the range of − 11 to − 19 per cent (columns 1 in Table 7), regardless of the measures used.^11^ Hence, our evidence suggests that a gender productivity gap exists and remains, even after controlling for many idiosyncratic sources of variation in productivity.

In order to look at Hypothesis 1, we next explore the role of finance constraints in explaining the observed gender gap. To do so, we include a variable for finance constraints and its interaction with the female variable in the regression equation

3 $${tfp}_{it}={\beta }_{1}{\mathrm{female}}_{i}+{\beta }_{2}{FC}_{i,t-1}+{\beta }_{3}{\mathrm{female}}_{i} \times {FC}_{i,t-1}+\gamma {X}_{it}+{\delta }_{s}+{\delta }_{l}+{\delta }_{t}+{\varepsilon }_{it}$$

Variable *FC* is a self-reported measure showing how important an enterprise ranks its access to finance from a list of 9 business constraints listed in the survey. *FC* can adopt integer values from 1 to 9, where a larger value indicates more serious finance constraints. To mitigate concerns over the simultaneity between productivity and finance constraints, we exploit the fact that *FC* is a time-varying variable and includes its first lag in the regression.

When *FC* is included but without any interaction term (columns 2 in Table 7), we find no significant relationship between productivity and the finance constraints reported a year earlier. When *FC* is interacted with *female* (columns 3 in Table 7), the estimate for ${\beta }_{2}$ remains statistically zero, while the estimate for the interaction term (${\beta }_{3}$) is found to be significantly negative at around − 0.07.

How do we interpret this finding in connection with our first hypothesis, H1, stating that within each gender group (female vs male), differences in funding explain productivity differences? Reading off the results for the interaction term and the female dummy, we can conclude the following: Males reporting financial constraints perform (statistically) no worse nor no better than those that do not. For females, this situation is different. Here, the better-funded female-owned businesses are associated with the highest productivity levels. Analogously, their weaker-funded peers perform significantly worse. Interpreting these findings, by looking across the gender category, financial constraints only seem to bind for the female-owned businesses (significance of the female × lagged finance covariate). More concretely, among female-owned enterprises, ranking finance constraints by one place higher (on a scale of 1 to 9) associates with an almost 7 per cent dip in productivity, all things equal. In other words, the negative female-to-male productivity gap increases with the severity of the finance constraints reported by females.

We can also express these findings in a different way. While self-reported finance constraints do not explain productivity differences between male-owned enterprises, they do so for female-owned ones. As a result, the negative female-to-male productivity gap increases with the severity of the finance constraints reported by females. Moreover, the inclusion of the interaction term seems to explain away the gender gap entirely, as the estimate for ${\beta }_{1}$ turns statistically insignificant and even positive in some regressions once the interaction term is included. This is in line with H1.

**Fig. 2:**
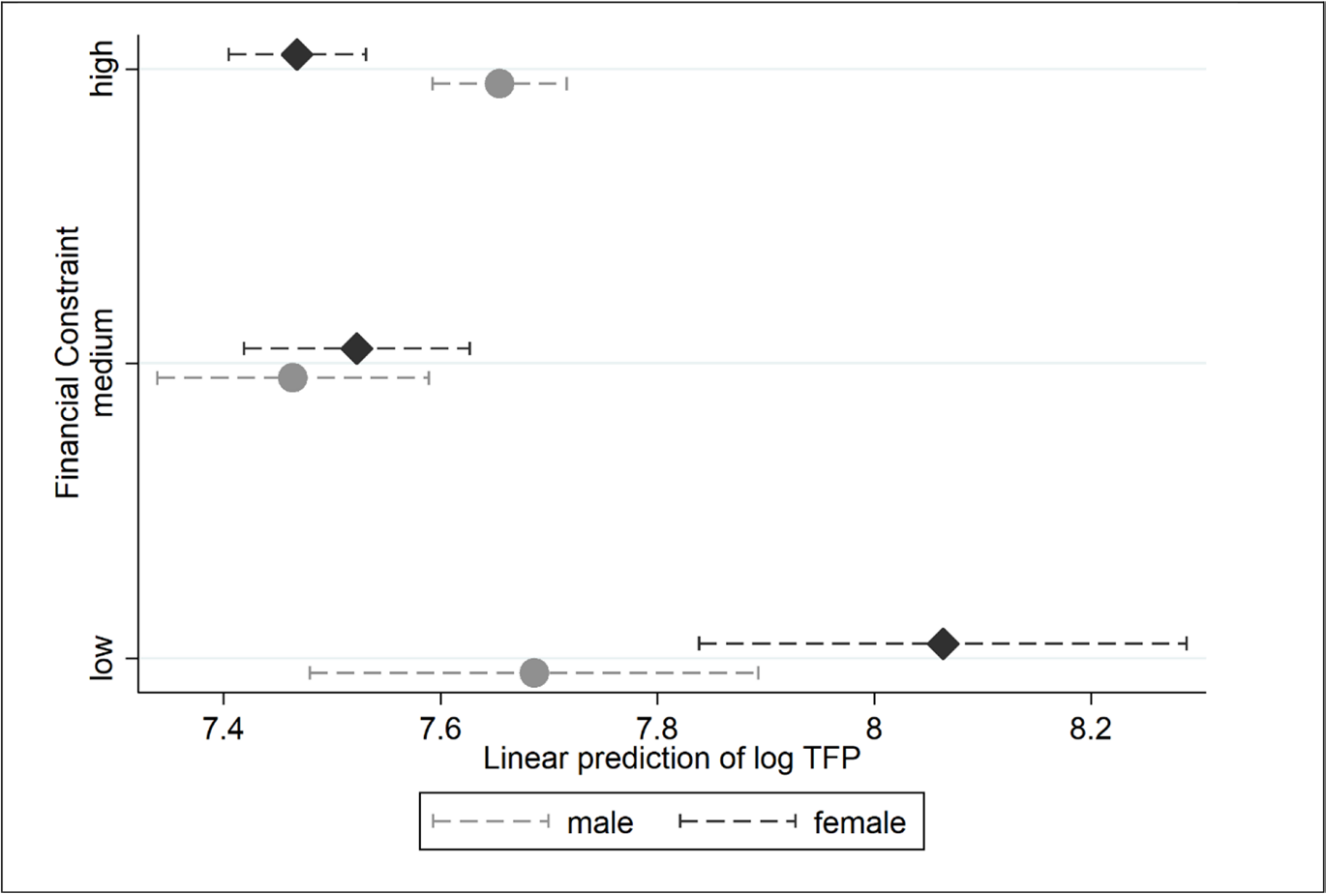
Gap in female-entrepreneur TFP and perceived financial constraints

Graphically, we can depict the main information from Table 7 how financial constraints underpin the predicted TFP of female- and male-owned businesses (Fig. 2).

In Fig. 2, the problem of gender-biased underfunding is thrown into sharp focus. The figure plots predictive margins from a regression in which the financial constraint variable is split into three binary variables: low, medium and high. A low financial constraint corresponds to values up to and including 3. For medium financial constraints, we consider values between 4 and 6 inclusive. And high financial constraints correspond to values above 6. Female business owners who categorise access to finance as relatively unproblematic (*financial constraint* = *low*) are associated with predicted TFP (black dotted line), outstripping that predicted for the male control group (grey-dotted line). The reverse is true for businesswomen whose business operations are most severely hamstrung by a lack of finance (*financial constraint* = *high*). Here, their similarly underfunded male peers are predicted to have higher TFP rates, all things equal. We can only hypothesise the reasons for this pattern – for the comparatively high TFP predicted for males versus females – when funding is restricted. Males may be able to tap alternative sources of funding – a possibility not open to females. Or males may be able to manage on a tighter budget, aligning the scale of their operations to match their funding. Whatever the reason, the TFP of female business owners responds most adversely to funding problems. The same is less true for male business owners.

### Different sources of finance

Next, we explore the relationship between productivity and the use of different finance sources in order to deal with Hypothesis 2. In the survey, enterprises are asked what percentage of their working capital was obtained from the following sources: bank loans, own resources, friends and relatives, supplier credit, equity and bond and others. Based on this information, we generate finance source variables (abbreviated as FS) that measure the percentage of working capital obtained from each of the above sources. The FS variables are time-varying, taking values between 0 and 100.

Simple pairwise correlations between the finance constraint variable (*FC*) and the finance source (*FS*) variables (Table 6) reveal that enterprises reporting more severe finance constraints typically use a greater portion of their own resources, and, in the case of female-owned businesses, resources from friends and relatives to finance their liquidity. In contrast, the use of bank loans, supplier credit and equities/bonds is associated with enterprises reporting less severe finance constraints.

To examine how the different finance sources correlate with the gender productivity gap, we repeat our baseline regression, with *tfp_ac* as our chosen productivity measure (Table 8).

**Table 8:** TFP, gender and finance sources

Dependent variable:	tfp_ac	tfp_ac	tfp_ac	tfp_ac	tfp_ac
Finance source:	Bank loan	Own resources	Friends and relatives	Suppliers credit	Equity and bond
Female	− 0.125**	0.0799	− 0.118**	− 0.143***	− 0.119**
	(0.0509)	(0.126)	(0.0513)	(0.0508)	(0.0506)
Lagged FS	0.000710	0.00165	− 0.00640***	− 0.00462**	0.000565
	(0.00206)	(0.00100)	(0.00211)	(0.00235)	(0.00285)
Female x Lagged FS	0.00492	− 0.00241*	− 0.000524	0.00644*	0.000864
	(0.00408)	(0.00139)	(0.00324)	(0.00336)	(0.00353)
Age of firm	− 0.00496**	− 0.00516**	− 0.00541**	− 0.00510**	− 0.00478**
	(0.00225)	(0.00228)	(0.00226)	(0.00225)	(0.00226)
Firm size categories (benchmark is micro)					
Small	− 0.0754	− 0.0721	− 0.0877*	− 0.0622	− 0.0706
	(0.0507)	(0.0508)	(0.0511)	(0.0504)	(0.0507)
Medium sized	0.0618	0.0793	0.0625	0.0904	0.0722
	(0.0919)	(0.0914)	(0.0922)	(0.0906)	(0.0919)
Foreign	0.00214	0.0102	− 0.0219	0.0729	− 0.0102
	(0.168)	(0.167)	(0.167)	(0.168)	(0.164)
Observations	2917	2917	2917	2917	2917
*R*-squared	0.090	0.091	0.095	0.091	0.090

Note: OLS estimation with industry, location and year effects. The dependent variable is tfp_ac, which is the logarithm of TFP estimated using the Ackerberg-Caves-Frazer method. Finance source variables are percentages of working capital financed from a given source. Firm size categories are micro (0–5 employees), small (6–19 employees) and medium (20 + employees). Robust standard errors in parentheses

^***^p < 0.01

^**^p < 0.05

^*^p < 0.1

Unlike the earlier regression, we now include the financial source (*FS* variables) in lieu of the financial constraints (*FC)* variables. The columns now report our regression results for different sources of finance – bank borrowing, own resources, financial support from friends and relatives, supplier credit, and equity and bonds.

Our regressions do not reveal strong associations between sources of finance and the gender productivity gap. The coefficients for the interaction terms in Table 8 are only statistically significant (at the 10 per cent level) in two cases. Two things are worth noting, however. First, the interaction term in the case of ‘own resources’ is significantly negative, indicating that the size of the gender-related productivity gap is more pronounced and the higher the share of own resources is for finance. This deterioration in the gender productivity gap, with an increased reliance of female business owners on their own savings as a source of funding, is consistent with the idea that female business owners face more severe difficulties than males in sourcing external finance. This finding ties in with our second hypothesis (H2), which conjectures that an overreliance on cash savings by female business owners is strongly connected to the gender productivity gap.

The second issue worth noting relates to supplier credit. Here, the positive and significant interaction term indicates that the gender gap seems to decrease with increased usage of supplier credit. This is potentially an interesting finding. While the use of supplier credit is associated with lower productivity among male-owned enterprises (as suggested by the significantly negative coefficient for lagged FS), this is not the case for female-owned enterprises. Female-owned businesses with higher usage of a supplier credit report a narrower productivity gap vs similar male-owned businesses. This result suggests that better access to supplier credits can play a role in levelling the playing field between credit-constrained male and female businesses.

### Exports

Our analysis now moves to the topic of exports, a metric of considerable interest to policymakers in LDCs. In concrete terms, we consider whether the enterprise is able to sell its products abroad. This section of our analysis maps to our third hypothesis (H3) that the gender exporting gap is driven by underfunded female-owned businesses.

Using maximum likelihood logit estimations, we explain the propensity to export, controlling for enterprise characteristics. Our ultimate aim is to shed light on the conjectured gender gap in exporting and the role finance constraints might play in this relationship.

Our dependent variable is a time-varying binary variable *exporter*, taking the value of 1 if an enterprise sells some of its output abroad and 0 otherwise. Finance constraints are measured by the financial constraint (FC) variable, which, for ease of interpretation, is again split into three categories (*FC is low*, *FC is medium*, and *FC is high*) as before.

Due to the small number of exporting enterprises in our data, we complement conventional logit estimations with penalized maximum likelihood logistic regressions. The conventional logistic regression tends to yield biased estimates when the occurrence of events (e.g. exporting among Ghanaian MSMEs) are rare events. The penalized logit, also known as a Firth Logit after its first application by Firth (1993), applies a correction to reduce the above bias.^12^

The marginal effect estimates from the exporting regressions are reported in Table 9. The estimates from the conventional logit and the Firth Logit are very similar, suggesting that the bias under the conventional method is not substantial.

**Table 9:** Gap in female-entrepreneur export participation and perceived financial constraints

Method:	ML logit			Penalized ML logit (Firth)		
	(1)	(2)	(3)	(1)	(2)	(3)
Dependent variable:	Exporter	Exporter	Exporter	Exporter	Exporter	Exporter
Female	− 0.0200**	− 0.0190**	0.0688	− 0.0214**	− 0.0203**	0.0648
	(0.0085)	(0.0085)	(0.0683)	(0.0089)	(0.0090)	(0.0568)
Lagged tfp_ac		0.0084***	0.0069**		0.0089***	0.0075**
		(0.0030)	(0.0030)		(0.0034)	(0.0034)
Lagged FC categories (benchmark is low)						
Lagged FC is medium			0.0221			0.0207
			(0.0190)			(0.0203)
Lagged FC is high			0.0189			0.0164
			(0.0152)			(0.0163)
Female × lagged FC categories						
Female × lagged FC is medium			− 0.0360			− 0.0350
			(0.0351)			(0.0335)
Female × lagged FC is high			− 0.1000***			− 0.1024***
			(0.0361)			(0.0338)
Age of firm	− 0.0004	− 0.0004	− 0.0002	− 0.0004	− 0.0003	− 0.0002
	(0.0004)	(0.0004)	(0.0004)	(0.0004)	(0.0004)	(0.0004)
Firm size categories (benchmark is micro)						
Small	0.0210**	0.0202*	0.0186*	0.0215*	0.0206*	0.0192*
	(0.0106)	(0.0104)	(0.0105)	(0.0114)	(0.0114)	(0.0114)
Medium sized	0.0730***	0.0727***	0.0693***	0.0768***	0.0765***	0.0734***
	(0.0130)	(0.0128)	(0.0122)	(0.0141)	(0.0140)	(0.0139)
Foreign	0.0400**	0.0462**	0.0501***	0.0440**	0.0507**	0.0557**
	(0.0177)	(0.0180)	(0.0172)	(0.0222)	(0.0222)	(0.0229)
Observations	2,298	2,298	2,298	2,298	2,298	2,298
Pseudo *R*^2^	0.136	0.146	0.176			
McFadden *R*^2^				0.149	0.161	0.195

Note: Maximum likelihood (ML) logit and penalized ML logit (Firth’s method) estimates. All regressions include industry, location and year dummies. Financial constraint categories are small (1 to 3), medium (4 to 6) and high (7 to 9). Firm size categories are micro (0–5 employees), small (6–19 employees) and medium (20 + employees). The table reports marginal effects. Standard errors are in parentheses and obtained with the Delta method. Robust standard errors for the logit regressions

^***^p < 0.01

^**^p < 0.05

^*^p < 0.1

We document a statistically significant gender gap in exporting. The marginal effect is − 0.02 (significant at a 5 per cent level), indicating that female-owned enterprises are 2 percentage points less likely to export than male-owned ones (columns 1 of Table 9). But because Ghanaian micro-enterprises export with a single-digit probability, even this small difference is economically meaningful. Moreover, the size of this gender gap remains virtually unchanged when controlling for lagged productivity (columns 2 of Table 9). Thus, the gender gap in exporting cannot be explained simply by gender differences in productivity.

**Fig. 3:**
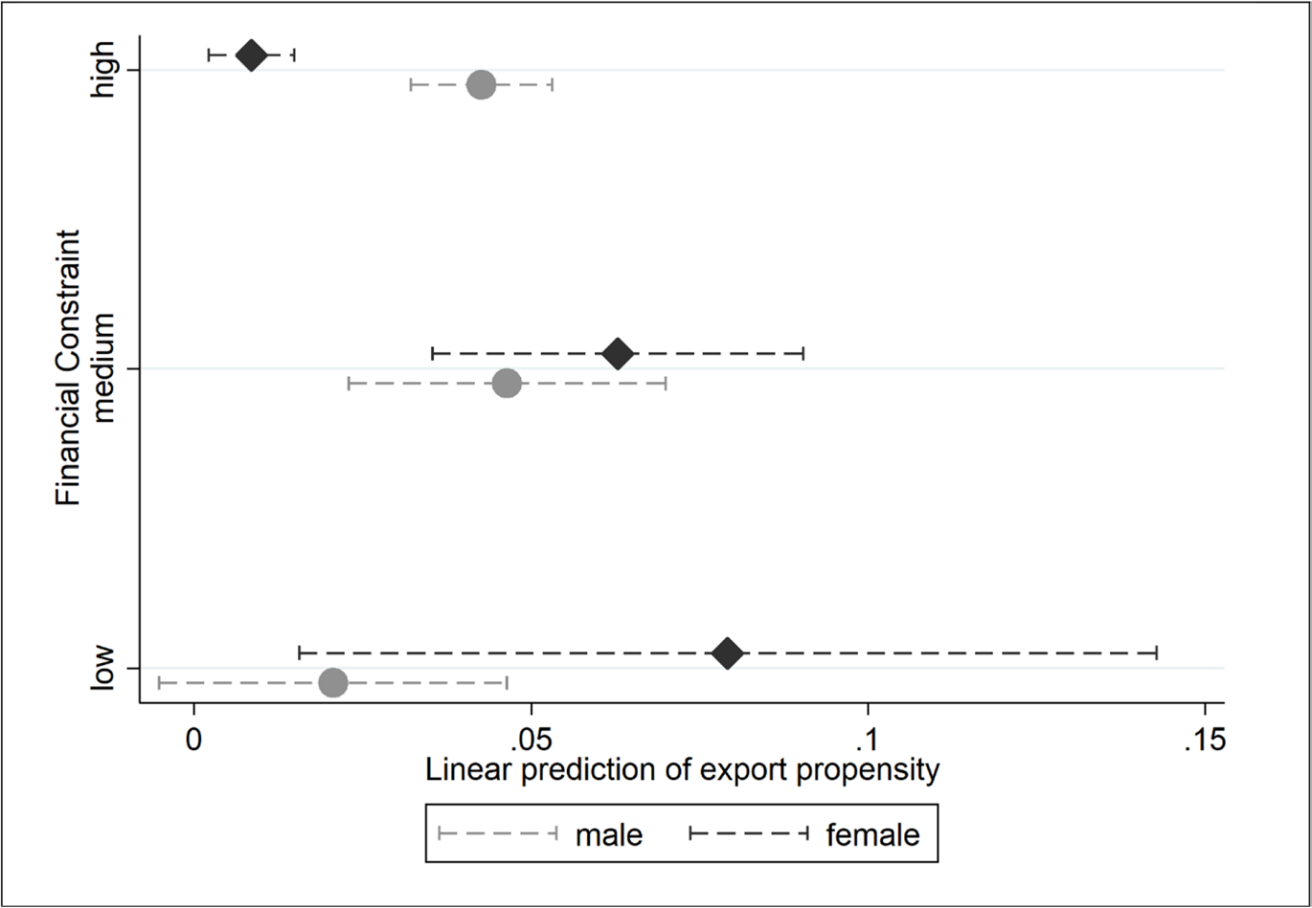
Gap in female-entrepreneur export participation and perceived financial constraints

Figure 3 illustrates the situation graphically. As the variable ‘financial constraint’ gets ranked from low to high, so too do female and male business owners differ in their propensity to export. Similar to the pattern for TFP, female business owners are predicted to have far higher export propensities when access to finance is less of a constraint. When finance is ranked as an exceptionally high problem, this export propensity falls to near zero.

Returning to our hypothesis concerning exports and gender-biased liquidity constraints, we can conclude that our consideration of the finance constraint indicators and their interactions with *female* suggests that the export gender gap is driven by those female-owned enterprises reporting high finance constraints. These enterprises are almost 10 percentage points less likely to export than female businesses reporting low finance constraints, whose propensity, in turn, does not differ from the propensity to export of male-owned businesses (columns 3 in Table 9).

## Conclusions

In this first comprehensive study of the funding-performance gap of Africa’s female business owners, we analyse the productivity and exports of females compared to their male peers. Since women are disproportionately active in economic sectors where businesses are traditionally smaller and more labour intensive (e.g. clothes manufacture), we control for the sector and other confounding variables which would otherwise skew our comparisons. Our estimation model builds on the intuition that funding supports ex post business performance, all things equal. Now, we view our funding-performance model through the lens of gender. Where comparisons of credit-unconstrained females outperform their male peers from the ‘unconstrained’ group, there is a case to be made that the more promising females are inadequately provided for by banks (market failure) since the marginal productivity of finance is higher for this subgroup.

In line with other studies, our estimations do indeed reveal a robustly significant gender performance gap. In terms of magnitude, we estimate the performance differential lies somewhere between 11 and 19 per cent, depending on the TFP measure used. Interestingly, the severity of the finance constraints reported by females is critical. This performance gap disappears when gender is interacted with the magnitude of funding constraints. On one end of the funding spectrum, there is no significant performance difference between males and females, where females receive adequate funding. But female business owners towards the higher end of the funding constraints scale (one placing higher on a scale of 1 to 9) report an almost 7 per cent productivity dip, all things equal. For male business owners (control group), there is no equivalent productivity dip, as financial constraints increase in severity. Perhaps males can divert funding from alternative sources. But for females, where household and business are likely to share a common budget, investing in her business can mean a zero-sum game.

An additional result connects to the source of funding – not all funding sources are created equal. For females reliant on their own savings, the absolute size of the gender gap is more pronounced. This negative connection between the use of savings by businesswomen and performance hints at a worrying possibility – Females reliant on their own resources to finance their business may be forced to do so from a lack of competitive alternatives.

On a more positive note, females using supplier credit report a narrower productivity gap compared to their male peers. This result suggests that better access to supplier credits can play a role in levelling the playing field between credit-constrained female- and male-owned businesses. Conversations with practitioners in Ghana have underscored the importance of this trust-based relationship between businesswomen and their suppliers. Supply credit can offer something of a lifeline – allowing women to align their cash receipts to their cash outgoings.

In terms of policy, we can derive a few conclusions. Policymakers are understandably wary about dictating to banks and other lenders, which lending criteria they should apply when judging between good and bad business risks. Only in the case of systemic market failure is there a prima facie case for taking a policy step. Our evidence hints that such market failure is indeed a possibility. Despite controlling for various characteristics of Ghanaian businesses, industry, location and year, the fact still remains that Ghanaian female business owners seem more seriously impeded by a lack of credit access than their male counterparts. For female business owners, this obstacle translates into compromised productivity and export potential.

As with any study of this kind, there are caveats. First and foremost, some of the phenomena examined are still very much marginal activities – what could be considered atypical for the average firm. Exporting is only pursued by 3–4 per cent of firms. Yet, exporting is considered a key avenue for building the most exceptional firms – those capable of contesting the global stage.

We now return to the original question of female business owners and our evidence of a funding gap. The question remains, how can this problem be tackled? Apart from an open confrontation with lenders – urging them to reconsider their lending strategies – there are other possibilities. These include greater recognition of the role of supplier credit in underpinning the liquidity of businesses. Tax concessions for suppliers which provide these credits might represent one avenue for supporting the liquidity of female-owned businesses. This policy would most likely help businesswomen at the middle-lower end of the performance distribution. A further possibility for helping the female business owners at the top end of the performance distribution (Africa’s Lionesses) is examining alternative funding structures – how best to target equity and bond packages towards these exceptional businesswomen. In this way, female business owners such as Bethlemen Tilahun Alemu, creator of the international footwear phenomenon *soleRebels*, could more easily ramp up their sales capacity, reduce their average costs and target foreign markets.

## Data Availability

For any questions regarding the data used (ISSER-IGC Panel), please e-mail Charles Ackah (ackah@ug.edu.gh).

## Footnotes

1 In a list of Middle East and North Africa (MENA) economies.

2 Of course, there are also other constraints which are not the focus of this paper. For example, Field et al. (2010) show that traditional cultural institutions in a country may put a constraint on female entrepreneurship.

3 To highlight the extent to which female business owners are credit constrained, Chaudhuri et al., (2020) employ a modified version of the Oaxaca (1973) technique.

4 Not all studies can be split into those reporting credit constraints, and those which do not. There is even one study, finding evidence of positive credit discrimination, where female-owned businesses are even preferred by banks (Hansen & Rand, 2014). Using data for 16 African countries and applying a method similar to Presbitero et al. (2014), Hansen and Rand demonstrate that small enterprises owned by females are less likely to be credit constrained compared to their male counterparts. An effect reversed for medium-sized businesses, where no such favouritism exists.

5 We should note that although neither Bardasi, Sabarwal & Terrell (2011) nor Gonzalez-Uribe & Leatherbee (2018) highlight differences between female and male applicants, they do suggest that type 2 selection error (rejection of promising candidates) is likely to be a problem – a point which underscores the need to look beyond evidence on loans, considering a broader palette of finance.

6 See Abeberese et al. (2019) for a detailed description and application of the panel. Because of the retrospective nature of the survey, a potential cause for concern is recall error. However, we should point out that data were collected through face-to-face interviews with respondents who were instructed to extract the information directly from the firm’s written records. In 60 per cent and 30 per cent of the interviews, the respondent was the owner or a senior manager, respectively. Furthermore, Abeberese et al. (2019) tested the robustness of their results by successively dropping earlier years from their estimation sample. Their estimation results remained robust to these modifications, suggesting that recall error does not seriously compromise the data.

7 Of possible productivity measures, we opt for total factor productivity (TFP) and capitalise on the recent developments in the econometrics of measuring TFP. As opposed to alternative productivity measures (e.g. labour productivity), TFP considers the efficiency of the use of all inputs in the production process. Moreover, thanks to available structural estimation methods, we are able to obtain an unbiased estimate for TFP, which accounts for the possible endogeneity of firm’s input decisions.

8 Note that we opt for a value-added production function (instead of a gross output production function with capital, labour and materials as inputs) because the simultaneous use of materials as proxy variable and as regressor in the production function estimation causes identification problems (Gandhi, Navarro and Rivers, 2020). Namely, production materials are assumed to be a flexible input, as is customary in the literature, and are used as the proxy variable for productivity..

9 A detailed description of this literature is provided by Rovigatti and Mollisi (2017), among others.

10 We perform the production function estimation using the prodest command in Stata, which was developed by Rovigatti and Mollisi (2017).

11 This percentage change is calculated as 100*exp(coefficient) − 100. The coefficient − 0.118 therefore converts to 11%, and the coefficient − 0.212 to − 19%.

12 To perform the penalized logit estimation, we use the firthlogit command in STATA, which was written by Joseph Coveney. Note that robust standard error estimation is not allowed by this command.
